# Genetic evaluation of relationship between mutations in *rpoB *and resistance of *Mycobacterium tuberculosis *to rifampin

**DOI:** 10.1186/1471-2180-9-10

**Published:** 2009-01-15

**Authors:** Anna Zaczek, Anna Brzostek, Ewa Augustynowicz-Kopec, Zofia Zwolska, Jaroslaw Dziadek

**Affiliations:** 1 Institute for Medical Biology, Polish Academy of Sciences, Lodz, Poland; 2 Department of Genetics, University of Rzeszow, Rzeszow, Poland; 3 Department of Microbiology, National Research Institute of Tuberculosis and Lung Diseases, Warsaw, Poland

## Abstract

**Background:**

Rifampin is a first line antituberculosis drug active against bacilli in logarithmic and stationary phase, which interferes with RNA synthesis by binding to bacterial RNA polymerase. Tubercle bacilli achieve resistance to rifampin by accumulation of mutations in a short-81 bp region of the *rpoB *gene. Among many mutations identified in the *rpo*B gene, few were verified by molecular genetic methods as responsible for resistance to rifampin (RMP).

**Results:**

In this study eight different mutations identified in an 81 bp section of a "hot spot" region of the *rpo*B gene of RMP resistant *Mycobacterium tuberculosis *clinical strains were evaluated in respect to drug resistance. It was found that: mutations in positions 526 (H/D), 516 (D/V) and 531 (S/L) result in high level resistance to rifampin; mutations in positions 516 (D/Y), 515 (M/I), 510 (Q/H) or a double mutation in codons 512 (S/I) and 516 (D/G) relate to low level of resistance. Gene *rpo*B carrying mutations in codon 513 (Q/L) introduced into an *M. tuberculosis *laboratory strain did not cause resistance to rifampin, however the same gene introduced into two different clinical strains did, with the level of resistance depending on the host strain.

**Conclusion:**

Mutations in an 81 bp "hot spot" region of the *rpoB *of *M. tuberculosis *lead to different levels of resistance to rifampin. Some mutations in this "hot spot" region of *rpoB *require a specific genetic background for the host strain to develop resistance to rifampin. Therefore, the identification of such mutations in a clinical *M. tuberculosis *strain is not enough to classify the given strain as resistant to rifampin.

## Background

Tuberculosis (TB) is a devastating infectious disease causing high mortality and morbidity worldwide with 8 million new TB cases and 2–3 million deaths annually. The situation of TB is made even worse by the rising emergence of drug resistant strains of *Mycobacterium tuberculosis*. Multi-drug resistant TB (MDR-TB) is defined as resistant to at least isoniazid (INH) and rifampin (RMP), the two most active first-line drugs against TB. MDR-TB treatment takes up to 2 years with second line drugs, which are expensive and have side effects. In 2006 US Centers for Disease Control and Prevention (CDC) and the World Health Organization (WHO) drew attention to the emergence of *M. tuberculosis *with extensive drug resistance to second-line antituberculosis drugs (XDR). XDR-TB is resistant to at least INH and RMP among the first-line drugs and to at least one of three injectable second-line anti-tuberculosis drugs used in TB treatment (capreomycin, kanamycin, amikacin) [1]. Thus, the treatment of such tuberculosis is becoming seriously limited, sometimes returning TB control to the pre-antibiotic era [1]. Tuberculosis chemotherapy started in 1944, when streptomycin (SM) was administered for the first time to a critically ill TB patient. Later, TB treatment was enriched with paraaminosalicylic acid (PAS-1949), INH (1952), pyrazinamide (PZA-1954), ethambutol (EMB-1962) and RMP (1963). It was identified that monotherapy generates drug-resistant mutants within a few months, endangering the success of antibiotic treatment. This problem was overcome by using combinations of drugs with as many as four drugs recommended nowadays by CDC and WHO [2].

The key antituberculosis drug commonly used in the treatment of tuberculosis is RMP. The loss of RMP as an effective drug leads to a need for a longer duration of therapy and often to a lower cure rate [3-6]. Drug resistance in *M. tuberculosis *is caused by mutations of various chromosomal genes, as identified for MDR occurrence due to the sequential accumulation of mutations in different genes that provide resistance to individual drugs. The individual molecular mechanisms of resistance have been identified for all first-line drugs and the majority of second-line drugs [7]. In *M. tuberculosis*, resistance to RMP results from mutations in the β-subunit of RNA polymerase, which is encoded by the *rpo*B gene [8]. Approximately 95% of RMP-resistant strains carry mutations within an 81-bp region containing codons 507 through 533 of the *rpoB *gene [8-10]. The single mechanism of resistance and narrow distribution of mutations make *rpoB*-81 bp region very attractive for molecular detection of resistance to RMP [11,12]. However, within several dozen different mutations detected in the *rpoB*-81 bp region of RMP-resistant *M. tuberculosis *strains [for review see [13]], very few were tested by cloning and complementation assays. Mutated *rpoB *genes (S531L; H526Y; D516V) were introduced into the RMP sensitive *M. tuberculosis *H_37_Rv strain, resulting in acquired drug resistance of the host strain [14]. These authors observed that the level of acquired resistance was higher for mutants carrying mutations in codons 531 and 526 compared to mutation in codon 516. In this paper a genetic model was constructed allowing for a relatively simple verification of the relationship between the presence of a given mutation in *rpoB*-81 bp region and the RMP resistance of the host strain carrying such a mutation. Some *rpoB *mutations revealed drug-resistance only in selected *M. tuberculosis *strains suggesting that genetic background of the host is important for the development of resistance to RMP.

## Methods

### Bacterial strains and growth conditions

The *M. tuberculosis *strains examined for this study were isolated from TB patients in Poland in 2000 during the second national survey of drug resistance [12,15]. Eight clinical strains identified as drug resistant, carrying different mutations in the *rpoB *gene, and two susceptible strains identified as drug sensitive, which did not carry any mutation in *rpoB*, were selected. Moreover, a control laboratory strain *M. tuberculosis *H_37_Ra, was included in this study. Primary isolation, differentiation, and drug susceptibility testing were performed with Lowenstein-Jensen (LJ) medium and the BACTEC 460-TB system (Becton-Dickinson, Sparks, Md.), as reported earlier [15]. All mycobacterial strains used in this study were cultured in Middlebrook 7H9 broth supplemented with OADC (albumin-dextrose-sodium chloride) and with kanamycin (25 μg/ml), or hygromycin (10 μg/ml), when required. Mycobacterial transformants were selected on Middlebrook 7H10 agar plates enriched with OADC containing kanamycin (Km) or hygromycin (Hyg).

### Gene cloning strategies

**Figure 1:**
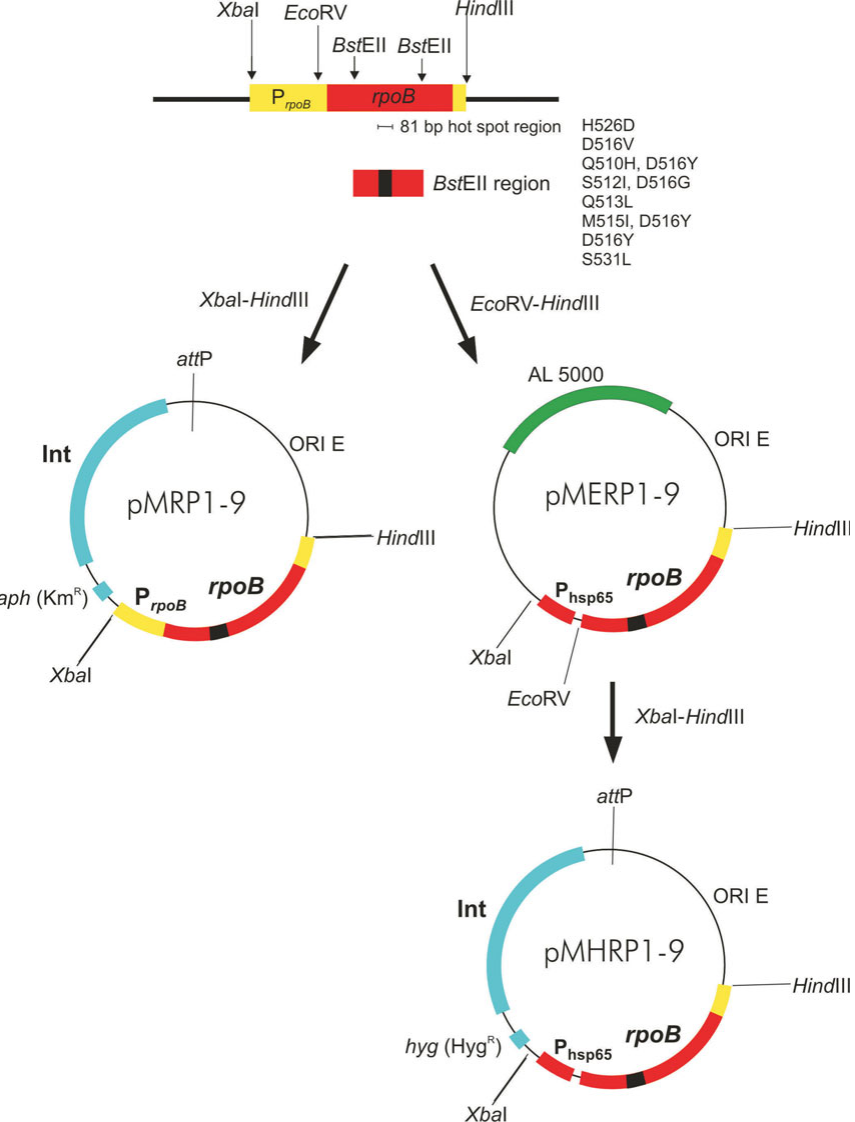
**Construction strategy of integration (pMRP1-9; pMHRP1-9) and self-replicating (pMERP1-9) plasmids carrying wild type and mutated *rpoB *genes under control of own (pMRP1-9) and heat shock (pMHRP1-9; pMERP1-9) promoter**. Description in the text.

Standard molecular biology protocols were used for all cloning procedures [16]. All PCR products were obtained using thermostable ExTaq polymerase (Takara) and cloned initially into pGemT vector (Promega), sequenced, and then released by digestion with appropriate restriction enzymes before cloning into the final vectors. Some restriction enzymes recognition sites were incorporated into the sequence of primers. The primers and plasmids used in this work are listed in Table 1 and 2, respectively. To engineer various *rpoB *genes of *M. tuberculosis *controlled by a natural promoter, a basal pRpoZero vector was constructed (Fig. 1). The vector contained the 5' end of *rpoB *until a natural *Bst*EII restriction enzyme recognition site (681 plus 950 bp of upstream region) which was connected to the 3' fragment of the gene starting with a natural *Bst*EII restriction enzyme recognition site (1122 plus 218 bp of downstream region). The resultant construct was used for cloning of the inner *Bst*EII-*Bst*EII fragment (1716 bp) of *rpoB *genes from various *M. tuberculosis *clinical strains resistant to RMP. The correct orientation of cloned *Bst*EII fragments was verified by digestion with *PvuII *endonuclease. Next, the cloned genes controlled by their natural promoter, carrying given mutations or wild type sequence in the hot spot region were relocated into the pMV306 integration vector. The resultant constructs (pMRP1-9) were electrotransformed into RMP susceptible strains, and the integration of DNA was monitored by Km selection and verified by PCR. Alternatively, the investigated *rpoB *genes were relocated without putative promoter sequence into pMV306P_*hsp *_integration vector under control of strong promoter (P_*hsp*65_). The resultant constructs (pMHRP1-9) were electrotransformed into RMP susceptible strains, and the integration of DNA was monitored by Hyg selection and verified by PCR.

**Table 1:** Primer sequences used for PCR amplification

Amplified region	Primer	Sequence	Product size (bp)
promoter region (950 bp) and 5' part of *rpoB *gene (721 bp)	P-rpo-s	5'-tctagacgagagcggcggtgcaatc	1671
	P-rpo-r	5'-gctcgctggtccagcccagc	
3' part of *rpoB *gene (1258 bp) and downstream region (218 bp)	3'rpo-s	5'-cgacaccaagctgggtgcgg	1476
	3'rpo-r	5'-aagcttccagtcgcgagtcggcccg	
*Bst*EII fragment of *rpoB *gene including 81-bp hot spot region	bst-s	5'-cgcgacaccgtcggcgtgcg	1852
	bst-r	5'-aagtgtcgcgcacctcgcgggc	
pMV306 (221 bp) and insert DNA cloned in MCS of this vector	MV-r	5'-aaggcccagtctttcgactgagc	221 + insert
	MV-s	5'-gtggataaccgtattaccgcc	DNA

**Table 2:** Plasmids used in this study

Plasmid	Description	Source
**Cloning vectors**		

pGemTEasy	T/A cloning	Promega
pMV306H	mycobacterial integrating vector, Hyg^R^	Med-Immune Inc.
pMV306K	mycobacterial integrating vector, Kan^R^	Med-Immune Inc.
pMV261	mycobacterial *Escherichia coli *shuttle vector, carrying heat shock (P_*hsp*65_) promoter, Km^R^	Med-Immune Inc.

**RpoB expression vectors**		

pMRP1	wild type *rpoB *of *M. tuberculosis *H_37_Ra controlled by natural promoter *P*_*rpoB *_cloned in integration vector pMV306K, Kan^R^	This study
pMRP2-9	mutated *rpoB *of *M. tuberculosis *clinical strains controlled by natural promoter *P*_*rpoB *_cloned in integration vector pMV306K; 2-represents H526D; 3-D516V; 4-Q510H/D516Y; 5-S512I/D516G; 6-Q513L; 7-M515I/D516Y; 8-D516Y; 9-S531L, respectively, Kan^R^	This study
pMERP1	wild type *rpoB *of *M. tuberculosis *H_37_Ra controlled by heat shock promoter P_*hsp*65 _in pMV261, Kan^R^	This study
pMERP2-9	mutated *rpoB *of *M. tuberculosis *clinical strains controlled by heat shock promoter P_*hsp*65 _in pMV261, 2-represents H526D; 3-D516V; 4-Q510H/D516Y; 5-S512I/D516G; 6-Q513L; 7-M515I/D516Y; 8-D516Y; 9-S531L, respectively, Kan^R^	This study
pMHRP1	wild type *rpoB *of *M. tuberculosis *H_37_Ra controlled by heat shock promoter P_*hsp*65 _in pMV306, Hyg^R^	This study
pMHRP2-9	mutated *rpoB *of *M. tuberculosis *clinical strains controlled by heat shock promoter P_*hsp*65 _in pMV306, '2-represents H526D; 3-D516V; 4-Q510H/D516Y; 5-S512I/D516G; 6-Q513L; 7-M515I/D516Y; 8-D516Y; 9-S531L, respectively, Hyg^R^	This study

### Susceptibility testing

Susceptibility testing was conducted using the proportion method on Youmans' liquid medium supplemented with 10% OADC with seven concentrations of RMP (50, 25, 12.5, 6.2, 1.5, 0.75, 0.37 μg/ml). The growth was determined after 21 days of incubation. The results were verified by Alamar Blue Assay [17-19] and by plating bacteria on Middlebrook 7H10 supplemented with OADC and various concentrations of RMP.

## Results

### The level of RMP resistance depends on the site and kind of substitution identified in the rpoB gene

The epidemiological studies carried out in many clinical laboratories worldwide have revealed several dozen mutations present in the *rpoB *gene of RMP resistant *M. tuberculosis *strains [12,14,20-23]. According to our knowledge, only three specific mutations of *rpoB *have been verified so far by molecular cloning techniques [14]. The complementation of RMP sensitive *M. tuberculosis *strain with *rpoB *gene carrying given mutation is not simply due to the gene length (3519 bp). One step amplification of gene together with its putative promoter based on *M. tuberculosis *genomic DNA as a template and its cloning is rather tough for investigators. To avoid this problem we have engineered pRpoZero vector carrying a 950 bp putative promoter region followed by 5'(721 bp) and 3' (1258 bp) *rpoB *gene fragments of an RMP-sensitive *M. tuberculosis *H_37_Ra strain (Fig. 1). The missing inner part of the *rpoB *gene flanked with natural *Bst*EII restriction sites contains an 81-bp mutable region. The *Bst*EII fragment (1716 bp) of *rpoB *gene can be easily amplified based on genomic DNA isolated from investigated *M. tuberculosis *RMP-resistant strains and cloned in frame to complete the *rpoB *gene in the pRpoZero system.

In this study we have selected eight *M. tuberculosis *RMP-resistant clinical strains carrying different mutations in *rpoB *gene [12] (Table 3). The PCR generated *Bst*EII inner fragments of the *rpoB *gene were verified by sequencing and were cloned into the pRpoZero vector. The correct orientation of insert was confirmed by *Pvu*II restriction analysis. Subsequently, the constructed genes, together with the putative promoter region, were relocated into the pMV306 integration vector using *Xba*I and *Hind*III restriction enzymes. The resultant constructs carrying wild type or mutated *rpoB *genes under control of a natural promoter, were electroporated into an RMP-sensitive *M. tuberculosis *H_37_Ra host. The integration of plasmid DNA into the *attB *site of chromosomal DNA was verified by PCR using MVs and MVr primers.

**Table 3:** Rifampin resistance of clinical and control *M. tuberculosis *strains

*M. tuberculosis *clinical strains	mutated amino acid of RpoB	MIC of rifampin (μg/ml)
Mt.2	H526D	25
Mt.3	D516V	25
Mt.4	Q510H; D516Y	25
Mt.5	S512I; D516G	12,5
Mt.6	Q513L	50
Mt.7	M515I; D516Y	25
Mt.8	D516Y	12,5
Mt.9	S531L	25
KL1936	-	1,5
KL463	-	1,5

**control strain**		

H_37_Ra	-	1,5

The wild type clinical strains and engineered *M. tuberculosis *H_37_Ra mutants were subjected to RMP-resistance analysis using the proportional method. Each strain was encoded by number and analyzed at least three times by standard procedure at the National Reference Center for Mycobacteria in Poland. The results obtained by the proportional method were verified using Alamar Blue Assay and by plating bacteria on Middebrook 7H10 supplemented with OADC and various concentrations of RMP (data not shown). The results obtained for clinical strains and engineered mutants are summarized in Table 3 and 4, respectively. Only three out of eight analyzed mutations (H526D; D516V; S531L) revealed the same level of RMP-resistance in clinical strains and engineered H_37_Ra mutants. Introduction of other mutations identified in RMP-resistant *M. tuberculosis *clinical strains into the H_37_Ra host did not result in resistance to RMP or the level of MIC was very low in comparison with clinical strains. Mutation of codon 516 substituting D with V resulted in a high level of RMP resistance. This effect was not observed when D was substituted with Y or G, even when an extra mutation was present in codon 510, 512 or 515.

**Table 4:** Rifampin resistance of *M. tuberculosis *recombinant clones

	MIC of rifampin (μg/ml) of *M. tuberculosis *recombinant clones carrying mutated *rpoB *gene controlled by:			
mutated amino acid of RpoB	*PrpoB*			*Phsp65*
	
	H_37_Ra	KL1936	KL463	H37Ra
H526D	50	50	50	50
D516V	25	25	25	25
Q510H; D516Y	1,5	6,2	6,2	6,2
S512I; D516G	6,2	6,2	6,2	6,2
Q513L	6,2	12,5	50	6,2
M515I; D516Y	6,2	6,2	6,2	6,2
D516Y	3,1	6,2	3,1	6,2
S531L	50	50	50	50

### Some rpoB mutations are able to cause RMP resistance only in a particular *M. tuberculosis *host

The observed different levels of resistance of *M. tuberculosis *clinical strains and H_37_Ra strain carrying *rpoB *genes mutated at the same positions lead to the conclusion that some mutations in the *rpoB *gene can reveal drug-resistant phenotype only in a specific genetic background of the host. To verify this hypothesis integration vectors carrying mutated *rpoB *genes under natural promoters were introduced by electroporation into two *M. tuberculosis *clinical strains (KL463; KL1936) sensitive to RMP. The selected transformants were verified by PCR amplification as described above. The resultant clinical strains carrying mutated *rpoB *genes were subjected to RMP resistance analysis by the proportional method. The results obtained were compared to the RMP-resistance of clinical strains carrying the same mutations and to the H_37_Ra recombinants described above (Table 4). The mutated *rpoB *genes generating high RMP-resistance level in *M. tuberculosis *H_37_Ra (H526D; D516V; S531L) were also responsible for high level of resistance of both clinical strains when introduced into their chromosomal DNA. On the other hand, mutation Q513L identified in an *M. tuberculosis *strain with resistance to a high level of RMP (MIC up to 50 μg/ml) which did not cause significant resistance of *M. tuberculosis *H_37_Ra (MIC up to 6.2 μg/ml), was responsible for RMP-resistance of KL463 and KL1936 strains at the level depending on the host (up to 12.5 and 50 μg/ml, respectively). The double mutation of *rpoB *in positions 510 (Q/H) and 516 (D/Y) identified in a highly resistant *M. tuberculosis *strain (MIC 25 μg/ml) which did not reveal resistance in H_37_Ra (MIC 1.5 μg/ml) was responsible for low level of resistance of both clinical tubercle bacilli hosts (MIC 6.2 μg/ml).

### The overproduction of mutated RpoB does not cause high level of resistance to RMP

We could not exclude that the different resistance of *M. tuberculosis *hosts carrying identical mutations in *rpoB *depends on different expression of RpoB controlled by unknown regulatory proteins. For example, the raised expression of target molecule (InhA) due to accumulations of mutations in promoter region is one of the known mechanisms of resistance to INH. As questions arose as to whether expression of mutated *rpoB *genes under control of the heat shock promoter (*P*_*hsp*60_) resulted in increased resistance of *M. tuberculosis *to RMP, the wild type *rpoB *and its mutated copies were cloned under control of the heat shock promoter as described in Methods. Although we did not have antibodies to test the level of expression for RpoB, the expression system is known to be very efficient [24,25]. The self-replicating constructs (pMERP1-9, Fig. 1) appeared to be very unstable when introduced into *M. tuberculosis *host (data not shown). Therefore the vectors (pMHRP1-9), which are able to integrate into *attB *site of mycobacterial chromosomal DNA, carrying wild type and mutated *rpoB *under *P*_*hsp*60 _promoter were constructed and electroporated into *M. tuberculosis *H_37_Ra. The presence of the relevant DNA introduced into the *attB *site of chromosomal DNA was verified by PCR amplification. The resultant recombinant strains were subjected to RMP resistance analysis by the proportional method. The results of RMP-MIC analysis obtained for strains carrying mutated *rpoB *genes under control of the strong *P*_*hsp*60 _promoter were similar to strains carrying the same *rpoB *genes under control of their natural promoter (Table 4). These observations suggest that the RMP-resistance of *M. tuberculosis *strains carrying *rpoB *mutated genes was not dependent on the *rpoB *expression level but resulted from the host genetic background that influence the drug-resistance phenotype.

## Discussion

All bacteria achieve resistance to RMP by mutations in a defined region of the RNA polymerase subunit β. In *M. tuberculosis*, approximately 95% of RMP resistant clinical isolates carry a mutation in the *rpoB *gene [8]. On the other hand, many isolates from *M. avium *and *M. intracellulare *present a natural resistance to RMP as a result of an efficient permeability and exclusion barrier [26,27]. Mutations in *rpoB *generally result in high level resistance to RMP. However, specific mutations in codons 511, 516, 518 and 522 can result in a lower resistance to RMP [14,28,29]. The role of some *rpoB *mutations (H526Y, S531L, D516V) in causing resistance was confirmed by genetic transformation experiments [14,30]. Several dozen other mutations identified in the *rpoB *gene of RMP-resistant *M. tuberculosis *clinical isolates have never been confirmed by genetic cloning [12,31-35]. Nowadays, when many genetic techniques are well developed, the knowledge about mutations connected to RMP-resistance is becoming used in the rapid identification of drug resistance [11,12,36,37]. However, the utility of these techniques depends on the precise information about the role of any given mutation in RMP resistance.

In this study we have engineered a genetic system which is helpful in the verification of the relationship between the presence of a given mutation in *rpoB *and RMP resistance. We have found that *rpoB *gene carrying either D516V or S531L mutation causes resistance to RMP when introduced into the *M. tuberculosis *hosts what was in agreement with previous investigations [14]. On the other hand, when mutated *rpoB *was introduced into drug sensitive *M. tuberculosis *laboratory or clinical strains, the other substitutions in position 516 (D/Y; D/G), even when supported with Q510H, M515I or S512I identified in RMP-resistant *M. tuberculosis *clinical strains, did not result in a significant increase of RMP-resistance. Other authors previously reported the identification of D516Y substitutions of *rpoB *in *M. tuberculosis *resistance to a high level of RMP [21,38], low level of RMP [14] and in strains sensitive to RMP [39]. Taken together, this suggests that D516Y/G substitutions in *rpoB *are not sufficient to result in RMP-resistance of *M. tuberculosis*. The substitutions in codon 526 (H/Y, D, R, L, P) were usually identified in *M. tuberculosis *clinical isolates highly resistant to RMP [14,23,38]. In this paper we have provided direct evidence that mutation H526D in *rpoB *is responsible for RMP-resistance when introduced into *M. tuberculosis *host. This result is similar to a previous finding verified by genetic transformation of *rpoB *carrying mutation H526Y [14]. By contrast, the contribution of *rpoB *carrying Q513L mutation to RMP-resistance was not that evident. The insertion of this gene into an *M. tuberculosis *H_37_Ra laboratory strain did not result in a significant level of RMP-resistance, however the insertion of the same gene was responsible for resistance to RMP of two *M. tuberculosis *clinical strains (MIC 12.5 and 50 μg/ml) when used as hosts. As identified in various clinical studies, the level of RMP-resistance of *M. tuberculosis *isolates carrying the Q513L mutation varies from 2 to 200 μg/ml [14,20,21,23,38]. The collected results suggest that *rpoB *carrying Q513L mutation is able to cause resistance to RMP only in selected tubercle bacilli. It is likely that this mutation can result in RMP-resistance in strains with low cell wall permeability since this exclusion barrier is responsible for natural resistance of some MAIC strains [26,27]. We also cannot exclude the possibility that other mechanisms support RMP-resistance of strains carrying Q513L mutation.

The drug resistance of *M. tuberculosis *can be also connected to the overproduction of a drug target due to accumulation of point mutations in a promoter region [40-42]. To test whether overproduction of *rpoB *carrying a given mutation result in higher MIC for RMP compared to a strain expressing the same gene under control of the natural promoter, *rpoB *genes were cloned under control of the *P*_*hsp *_promoter and introduced into *M. tuberculosis *host. The *P*_*hsp *_promoter, commonly used in genetics studies of mycobacteria controlling the *groEL *gene (Rv0440) in *M. tuberculosis*, has already been reported as highly active in mycobacterial cells growing *in vitro *[24,25]. A recent microarray study showed that the expression level of *groEL *in *M. tuberculosis *cells growing in log phase is high, but not higher than *rpoB *[43]. However, the arresting of *M. tuberculosis *growth results in 3.6-fold induction of *groEL *with a decrease of *rpoB *expression in the same conditions [44]. We have not observed higher RMP resistance when mutated *rpoB *genes were expressed under control of *P*_*hsp *_promoter in comparison to the natural promoter. It is possible that the natural level of RpoB is high enough to saturate RMP (if its concentration in cell is low). On the other hand, the extra expression of *rpoB *cannot help in cells accumulating high RMP level. However, to elucidate this problem an alternative expression system and precise control of protein expression would be required.

The natural resistance to RMP in some *M. avium *and *M. intracellulare *strains is known to be as a result of an efficient cell wall permeability and exclusion barrier [26,27], suggesting that these elements may be also important in *M. tuberculosis*. Changes in cell wall composition could affect permeability [45] decreasing the intracellular concentration of drug.

## Conclusion

Among several dozen amino acid substitutions identified in an *rpoB *81-bp region only a few are directly responsible for *M. tuberculosis *resistance to rifampin. Many others require a specific genetic background to develop resistance. Our findings lead to the conclusion that direct, molecular identification of rifampin resistant *M. tuberculosis *clinical isolates is possible only for strains carrying selected mutations in RpoB. The identification of other mutations suggests that investigated strains might be resistant to this drug.

## Authors' contributions

AZ performed the majority of experiments. AB helped in cloning. EAK and ZZ supervised susceptibility tests. JD conceived and supervised the study and wrote the manuscript. All authors have read and approved the final version of the manuscript.
